# Lack of CD34 delays bacterial endotoxin-induced lung inflammation

**DOI:** 10.1186/s12931-021-01667-2

**Published:** 2021-02-25

**Authors:** Gurpreet K. Aulakh, Sushmita Maltare, Baljit Singh

**Affiliations:** 1 Small Animal Clinical Sciences, Western College of Veterinary Medicine, University of Saskatchewan, Saskatoon, SK Canada; 2 Veterinary Biomedical Sciences, Western College of Veterinary Medicine, University of Saskatchewan, Saskatoon, SK Canada

## Abstract

**Background:**

CD34, a pan-selectin binding protein when glycosylated, has been shown to be involved in leukocyte migration to the site of inflammation. However, only one report is available on the expression and role of CD34 in neutrophil recruitment during acute lung inflammation.

**Methods:**

We proceeded to study the role of CD34 in lung neutrophil migration using mouse model of endotoxin induced acute lung inflammation and studied over multiple time points, in generic CD34 knock-out (KO) strain.

**Results:**

While there was no difference in BAL total or differential leukocyte counts, lung MPO content was lower in LPS exposed KO compared to WT group at 3 h time-point (*p* = 0.0308). The MPO levels in CD34 KO mice begin to rise at 9 h (*p* = 0.0021), as opposed to an early 3 h rise in WT mice (*p* = 0.0001), indicating that KO mice display delays in lung neutrophil recruitment kinetics. KO mice do not loose endotoxin induced lung vascular barrier properties as suggested by lower BAL total protein at 3 h (*p* = 0.0452) and 24 h (*p* = 0.0113) time-points. Several pro-inflammatory cytokines and chemokines (TNF-α, IL-1β, KC, MIP-1α, IL-6, IL-10 and IL-12 p70 sub-unit; *p* < 0.05) had higher levels in WT compared to KO group, at 3 h. Lung immunofluorescence in healthy WT mice reveals CD34 expression in the bronchiolar epithelium, in addition to alveolar septa.

**Conclusion:**

Thus, given CD34′s pan-selectin affinity, and expression in the bronchiolar epithelium as well as alveolar septa, our study points towards a role of CD34 in lung neutrophil recruitment but not alveolar migration, cytokine expression and lung inflammation.

## Background

Acute lung injury is characterized by an increase in permeability of the alveolar-capillary barrier, edema, and infiltration of neutrophils in the lung. Acute lung injury is marked by increased mortality (40%) due to alveolar infiltrates comprising of activated neutrophils, chemotactic peptides, pro-inflammatory cytokines and damaging mediators such as reactive oxygen species, and granule proteins [1–5]. While recruitment of activated neutrophils into inflamed tissues is critical for the defense of the organ, these activated cells also cause excessive tissue damage. Therefore, we need to devise ways to fine-tune the recruitment of neutrophils into the inflamed organs.

Leukocyte migration is a multi-step process composed of steps such as capturing, rolling, adhesion, activation, and movement across the endothelial [6, 7]. The cytokines, chemokines and expression of adhesive proteins such as selectins and integrins on endothelium and immune cells leads to recruitment of neutrophils in inflamed tissues [8–10]. The typical steps of neutrophil rolling, adhesion and transmigration are not well characterized in the lungs largely because of the challenges of application of intravital microscopy. Neutrophil migration in the lungs is different from the generally accepted leukocyte recruitment paradigm for several reasons. First, the narrow diameter and twisting nature of the lung capillaries result in retention of a large number of neutrophils in the capillaries [11–13]. Second, the pulmonary vasculature is a lower blood pressure region compared to the peripheral vasculature. Finally, depending upon the microbial stimulus for the inflammation, the recruitment appears to be independent of selectins and β2-integrins due to equivocal reports [12–14]. Currently, there is a need to identify additional molecules that regulate recruitment of neutrophils into the lung and their migration across the alveolar blood-air barrier.

CD34 belongs to the CD34-family of proteins and is a widely expressed sialomucin that is preferentially glycosylated in the high endothelial venules [15–17]. These proteins such as podocalyxin play an important role in cell shape change [18, 19]. Forced expression of CD34 induces cell rounding, microvillus formation, and phosphorylation of ezrin/radixin/moesin proteins in HEK293T cells while inhibiting integrin-mediated cell re-attachment to HEK 293 T cells [18, 20]. Platelets have been shown to bind to anti-CD34-coated expanded polytetrafluoroethylene vascular grafts [21]. The contribution of CD34 in lymphocyte tethering and rolling has been shown in vitro using laminar flow assays [22]. Upregulation of endothelial cell adhesion molecules, E-selectin and intercellular adhesion molecule 1 (ICAM-1) in response to interleukin-18 (IL-18), interferon-γ (IFN-γ), or tumor necrosis factor-α (TNF-α), is accompanied by downregulation of endothelial cell CD34 expression at mRNA as well as protein level. CD34 has thus been implicated in the negative control of endothelial adhesion due to this reversed pattern of expression [20]. CD34 reportedly binds all three selectins namely L-selectin (CD62L), E-selectin (CD62E) and P-selectin (CD62P) [18, 23–25]. CD34 facilitates inflammation by affecting neutrophil recruitment in the intestines of mice infected with *Salmonella typhimurium* [26]. CD34 KO mice show defective splenic dendritic cell chemotaxis [27] and pulmonary eosinophil migration [28], and are thus protected from contact hypersensitivity pneumonitis as well as Th2-dependent allergic asthma, respectively. Recently, Lo and colleagues showed that mice lungs lacking CD34 expression on the non-hematopoietic cells demonstrated higher sensitivity to bleomycin-induced lung injury [29].

Nevertheless, to our knowledge, only one report is available on the expression and role of CD34 in neutrophil recruitment during acute lung inflammation [29]. Considering the role of CD34 in cell shape and attachment [18, 20], and its role in leukocyte recruitment in allergic [28] and hypersensitivity models [27], we hypothesised that CD34 KO mice will show altered onset of vascular permeability, endothelial/leukocyte adhesion, and thus, transmigration in response to intranasal endotoxin (Lipopolysaccharide, LPS) compared with the WT mice.

## Materials and methods

### Animals

Ten- to twelve-week-old C57BL/6 WT mice were purchased from Charles River (Montreal, QC, Canada). CD34 knock-out (KO) mice were received from Dr. McNagny lab at University of British Columbia and were bred at the Lab Animal Service Unit (LASU, University of Saskatchewan, SK, Canada). Age-matched mice were used in all experiments. All experiments were approved by University Committee on Animal Care and Supply (UCACS) and Animal Research Ethics Board (AREB) of the University of Saskatchewan (Saskatoon, SK, Canada).

In this two factor experimental design, mice phenotype had two levels (WT and CD34KO) and time point had five levels (0, 3, 9, 15 and 24 h post-LPS treatment). Each of these ten groups has five-seen mice. Both male and female mice were equally divided across groups. Sample size calculation was performed using OpenEpi online tool (http://www.openepi.com/), with two-sided confidence level (i.e. 1-α) set at 95% and power (i.e. 1-β) set to 80%. The calculation yielded a sample size of 8–13 animals/phenotype (for each time-point), based on a minimum effect-size of two for any parameter. Please refer to Additional file 1: Fig. S1 for a schematic of this experiment design.

### LPS-induced lung inflammation

#### Experimental set-up

We used the direct intranasal LPS model in which an initial strong local, but not systemic, nasal and lung inflammatory response is followed by mobilization of the systemic compartments as well. Mice aged 12–16 week were anesthetized by isoflurane inhalation, and LPS (from *Escherichia coli* O55:B5; Sigma-Aldrich, Oakville, ON, Canada) diluted in sterile saline (1 g/L) was administered intranasally (50 µL /mice). Control mice at 0 h time point did not receive LPS. After 3, 9, 15 or 24 h, mice were sacrificed by cardiac puncture under deep terminal anesthesia induced by isoflurane (Additional file 1: Figs. S1). Blood was collected in acid citrate dextrose containing tubes. The blood (100 µL) from each non-coagulated sample was submitted to Prairie diagnostic services, Saskatoon, Canada for analysis of total leukocyte counts (TLC) and differential leukocyte counts (DLC). Plasma from rest of the blood sample was stored at − 80 °C.

Trachea was exposed through a midline incision and a sterile 23-gauge lavage tube was cannulated. Bilateral Broncho-alveolar lavage (BAL) was performed by instilling 0.5 ml sterile saline, thrice. The right bronchus was ligated at tracheal bifurcation to avoid protein denaturation in the right lung lobes and preserve them for myeloperoxidase (MPO) quantification. The left lung was perfused with cold saline to get rid of circulating blood, fixed via tracheal intubation with 4% paraformaldehyde and 0.1% glutaraldehyde and paraffin embedded. Right lung was flash frozen in liquid nitrogen and stored in − 80 °C for MPO estimation to determine lung neutrophil migration. The frozen right lung tissue (using liquid nitrogen), BAL supernatant and plasma were stored at − 80 °C until further analysis.

### BAL cellular analysis

BAL cytospin preparations were stained with Giemsa method to obtain BAL differential cell counts. The BAL total and differential counts were calculated and reported as cells/ml of blood. BAL supernatant was stored at − 80 °C for cytokine analysis using ELISA.

### Myeloperoxidase assay

Myeloperoxidase (MPO) estimation was performed to indirectly quantify the number of neutrophils left behind in the lung tissue after the broncho-alveolar lavage. The right mouse lung tissue, stored at − 80 °C, were mechanically homogenized in 500 μL of 50 mM HEPES (pH 8.0). Supernatant was discarded after centrifugation (10,000 g, 20 min) and the pellet was resuspended in 500 μL of 0.5% cetyl trimethyl ammonium chloride. Resuspended pellets were homogenized, centrifuged, and the resultant supernatant was aliquoted into new tubes. A standard curve was plotted by using standard known concentration of MPO from human leukocytes (M6908- 5UN; Sigma-Aldrich). Samples (10 µl) were pipetted into separate wells of 96-wells microtiter plates in duplicates followed by 65 μL phosphate citrate buffer. Lastly, 100 μL of 3,3′,5,5′-tetramethyl benzidine substrate was added and samples were incubated for 5 min to allow color development. After incubation, 150 μL stop solution was added to each well and absorbance were measured at O.D. 450 nm with a spectrophotometer.

### Immunofluorescence staining for CD34, Gr1 and von Willebrand Factor

Immunofluorescence (IF) was performed to localize the neutrophil marker, Gr1 and endothelial and platelet marker, vWF (Von Willebrand Factor), in WT and CD34 KO mice across different time-points. Tissue sections were deparaffinized and rehydrated followed by inactivation of endogenous peroxidase activity with 0.5% H_2_O_2_ in methanol in dark for 20 min at room temperature. After washing, antigen retrieval was performed in two steps. Firstly, heat induced epitope retrieval was done by incubating sections in sodium citrate buffer (10 mM, pH 6.0) at 90–95 °C for 20 min. Then, the sections were allowed to cool in distilled water, following which the sections were incubated with warmed pepsin (2 mg/ml in 0.01 N HCl) at 37 °C for 20 min. After washing, the sections were blocked with 1% bovine serum albumin in PBS for 30 min at room temperature and then incubated overnight at 4 °C with 100 µL primary antibodies per section against rabbit vWF (1:300 dilution, Catalogue A0082, Dako Denmark A/S, Glostrup, Denmark), rat Gr-1 (1:100 dilution; Catalogue 550,291; BD Biosciences, ON, Canada), and the sections were incubated overnight at 4 °C with 100 µL primary antibodies per section against CD34 (rabbit anti-mouse, EP373Y, ab81289, 1:500, Abcam Inc., Cambridge, MA, USA). Next day, the slides were left at room temperature for 30 min followed by washing with PBS 1X thrice. After washing, they were incubated in dark with 100 µL secondary antibody per section (AF488, green) for 30 min at room temperature, counterstained with DAPI, and dried. The cover slips were mounted with Prolong gold mounting medium, sealed with nail varnish and stored in dark at 4 °C.

### Lung vWF and Gr1 image analysis

Immune-fluorescent lung images from the above mentioned vWF, Gr1 and DAPI stained sections were evaluated for their staining and hence expression characteristics, using the open software Fiji ImageJ (https://imagej.net/Fiji). Fluorescence intensities (FI), skewness, kurtosis for Gr1, vWF and DAPI were quantified for WT and CD34 KO mice at 0, 9 and 24 h post-LPS exposure. After thresholding several lung regions in a merged field of view, the ratios of either Gr1 or vWF and DAPI arbitrary FI units (a.u.) were plotted, which are referred to as DAPI normalized FI units. Similarly, perimeter normalized FI, skewness or kurtosis were quantified for Gr1 and vWF by dividing them with the lung perimeter (in μm) of the regions of interest. A minimum of 3–5 regions were quantified from each image and all the regions from N = 2 per group were pooled to represent data-points for the 0, 9 and 24 h groups.

### Lung histology

Four-micrometer sections prepared from paraffin-embedded lung tissues were stained with Hematoxylin and eosin (H&E) stain and analyzed by a pathologist who was blinded for groups. Lung perivascular, peribronchiolar and septal inflammation and damage was analyzed and semi-quantitatively scored. Inflammation levels were scored based on congestion, neutrophil infiltration, and endothelial damage. The higher level of congestion, endothelial damage and neutrophil infiltration correspond to a higher score for overall inflammation. Absence of inflammation was recorded as “0”, minimal inflammation as, “1”, moderate inflammation as, “2” and intense inflammation as, “3”.

### BAL total protein estimation assay

Total protein concentration estimation in the BAL supernatant was performed using a colorimetric assay, DC™ Protein Assay (Bio-Rad, DC™ Protein Assay, California, USA). The amount of protein in the BAL supernatant is an indirect measure of vascular permeability of the lungs. Standard or samples (5 µl each) of BAL fluid were pipetted into separate wells of 96-wells microtiter plates in duplicates. This was followed by adding 25 µl of Reagent A’ (1 mL alkaline copper tartrate solution Reagent A + 20 µl surfactant solution Reagent S) to each well. Lastly, 200 µl of Reagent B (dilute Folin reagent) was added to each well. The samples were incubated for 15 min and the absorbance was measured at 750 nm by spectrophotometer.

### Broncho-alveolar lavage cytokine analysis

Cytokine were quantified to characterize the differences in LPS induced inflammation among WT and CD34 KO mice. Cytokine levels were studied in BAL fluid supernatant using commercially available multiplex ELISA kit, Bio-Plex Pro™ Mouse Cytokine 23-plex Assay (#m60009rdpd, Bio-Rad, California, USA). The cytokines and chemokines included in the panel are TNFα, IL-6, IL-1β, MCP-1, KC, IL-10, IL-1α, IL-2, IL-3, IL-4, IL-5, IL-9, IL-12 p40, IL-12 p70, IL-13, IL-17A, G-CSF, GM-CSF, IFN-γ, MIP-1α, MIP-1β, RANTES, and Eotaxin. All procedures were carried out according to the manufacturer’s instructions. Samples were loaded in duplicates, and signal detection was done using the Luminex Bio-Plex 200 system.

### Statistical analyses

Statistical analysis was performed using GraphPad Prism software version 8 (San Diego, CA, USA). Quantitative results were expressed as median and error bars represented standard error. Normal distribution of residuals was tested by histogram and Shapiro–Wilk test. The logonormal datasets (as per the Shapiro–Wilk test) from 2 × 2 experiment design were subjected to logarithmic transformation, application of two-way ANOVA (Analysis of Variance) followed by Dunnett’s and Sidak’s multiple comparisons tests which allow for corrections of any random effects arising out of the variables of genotype or time-point. The critical value of α was set to 0.05 as a significant difference (two-tailed).

## Results

### CD34 KO mice display subtle differences in the kinetics of blood leukocyte counts post-LPS treatment

**Fig. 1:**
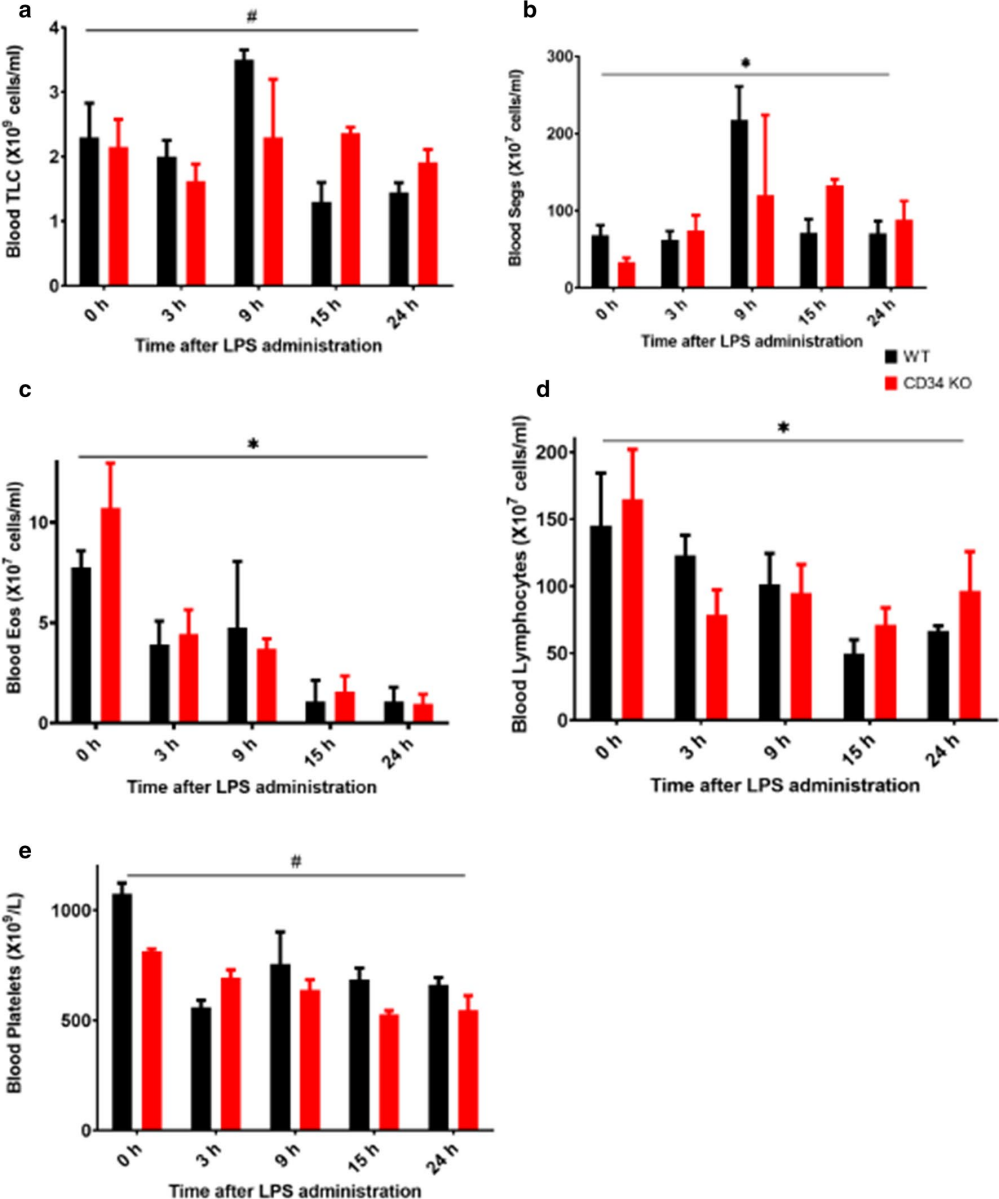
** a**–**e**: Peripheral blood cell counts at 0-h (no LPS; WT, N = 3; CD34 KO, N = 4), 3 h (WT, N = 5; CD34 KO, N = 4), 9 h (WT, N = 3; CD34 KO, N = 2), 15 h (WT, N = 3; CD34 KO, N = 3) and 24 h (WT, N = 6; CD34 KO, N = 6) post-LPS treatment. Results are expressed as mean ± SE. **a**Blood TLC; **b** Blood Segs; **c** Blood Eos; **d** Blood Lymphocytes; **e** Blood Platelets. ^#^Indicates significant interaction effect and *Shows significant main effect of time. No group differences were observed for any of the time points. *P* < 0.05

Cell counts were performed on the blood samples to study if lack of CD34 had an effect on peripheral blood counts (Fig. 1a). Two-way ANOVA of TLC show that interaction effect was significant (p = 0.04), strain were borderline (p = 0.05) while LPS effects were not significant (p > 0.05). However, as shown in Fig. 1a, no difference was observed between WT and CD34 KO mice at any of the time-points. Segmented neutrophils (Seg) in blood samples analyzed with two-way ANOVA (Fig. 1b) showed main effect of time-point post-LPS challenge was significant (p = 0.033). Other than significant increase in segmented neutrophils CD34 KO mice at 15 h time point compared to CD34 KO at 0 h, no other differences were found. While there were time effects within strain for eosinophils (*p* < 0.001, Fig. 1c), lymphocytes (*p* < 0.05, Fig. 1d) and platelets (*p* < 0.0001, Fig. 1e), there were no differences for these cells types between normal or LPS treated WT and CD34 KO mice.

### CD34 KO mice show delays in LPS-induced lung neutrophil recruitment when compared to WT mice

**Fig. 2:**
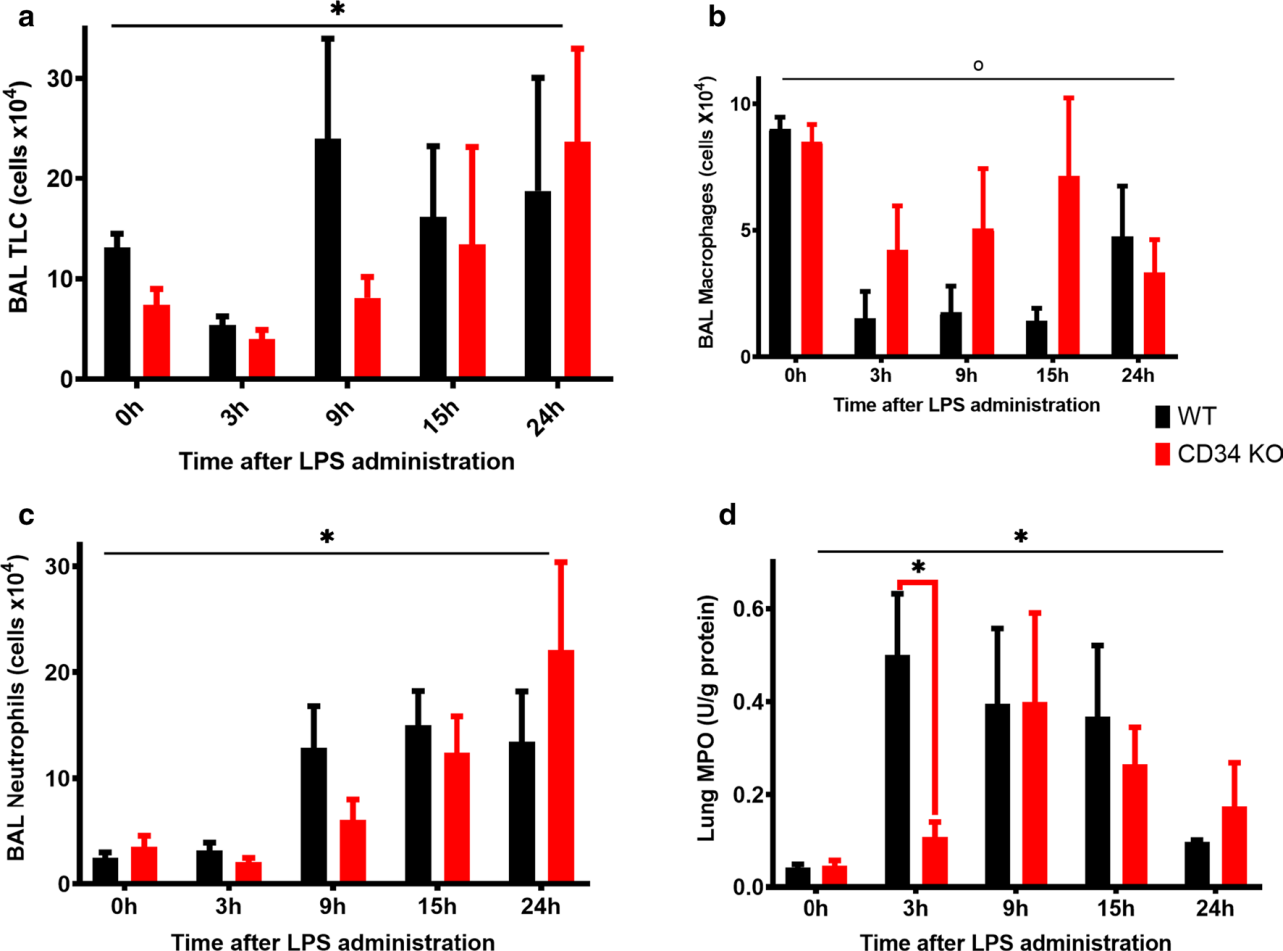
**a**–**d** Bronchoalveolar Lavage Fluid (BAL) and lung MPO at 0-h (no LPS; WT, N = 7; CD34 KO, N = 7), 3 h (WT, N = 6; CD34 KO, N = 6), 9 h (WT, N = 6; CD34 KO, N = 5), 15 h (WT, N = 3; CD34 KO, N = 3) and 24 h (WT, N = 6; CD34 KO, N = 6) post- LPS treatment with 50 μg LPS. Results are expressed as mean ± SE. **a** BAL Total Leukocyte Counts; **b** BAL Macrophages; **c** BAL Neutrophils; **d** Lung Myeloperoxidase (MPO) amount per gram of lung protein and experiment was performed in duplicates. *Indicates significant interaction effect, and °Indicates main effect of genotype. Significant group differences are indicated by bridged bars carrying *P < 0.05

Dunnett’s multiple comparisons test revealed no significant difference between the TLC at any of the post-LPS administration time-points in WT mice compared with CD34 KO mice (*p* > 0.05, Fig. 2a). Intranasal LPS instillation induced a significant (*p* < 0.01) time-dependent broncho-alveolar leukocyte recruitment, in both WT and CD34 KO mice (Fig. 2a).

Next, we looked at the total macrophage counts in BAL (Fig. 2b). A two-way ANOVA revealed that significant main effects of time-point post-LPS challenge (*p* < 0.01) and mouse strain (*p* = 0.04) on macrophage numbers. However, interaction effect was non-significant (*p* = 0.15). The intranasal instillation of LPS induced no difference between WT mice and CD34 KO mice at any of the time-points post-LPS administration (*p* > 0.05) (Fig. 2b).

A two-way ANOVA of neutrophil numbers (Fig. 2c) revealed no interaction effect (*p* = 0.45); but the main effect of time-point post-LPS challenge was significant (p < 0.01) underscoring that intranasal LPS instillation induced time-dependent changes in overall neutrophil counts. There however were no differences observed between the number of neutrophils in the BAL fluid of WT and CD34 KO mice at any of the time-points post-LPS administration (Fig. 2c). CD34 KO mice had 6.3 fold more BAL neutrophils at 24 h when compared to 0 h (*p* = 0.02) (Fig. 2c).

MPO estimation performed to quantify the number of neutrophils trapped in the lung (Fig. 2d) showed a significant (p < 0.01) main effect of time-point post-LPS challenge and both the strains showed increased MPO measurements compared to 0 h. Only at 3-h post-LPS challenge, the MPO content was significantly higher in WT mice compared with CD34 knock-out mice (*p* = 0.03).

**Fig. 3:**
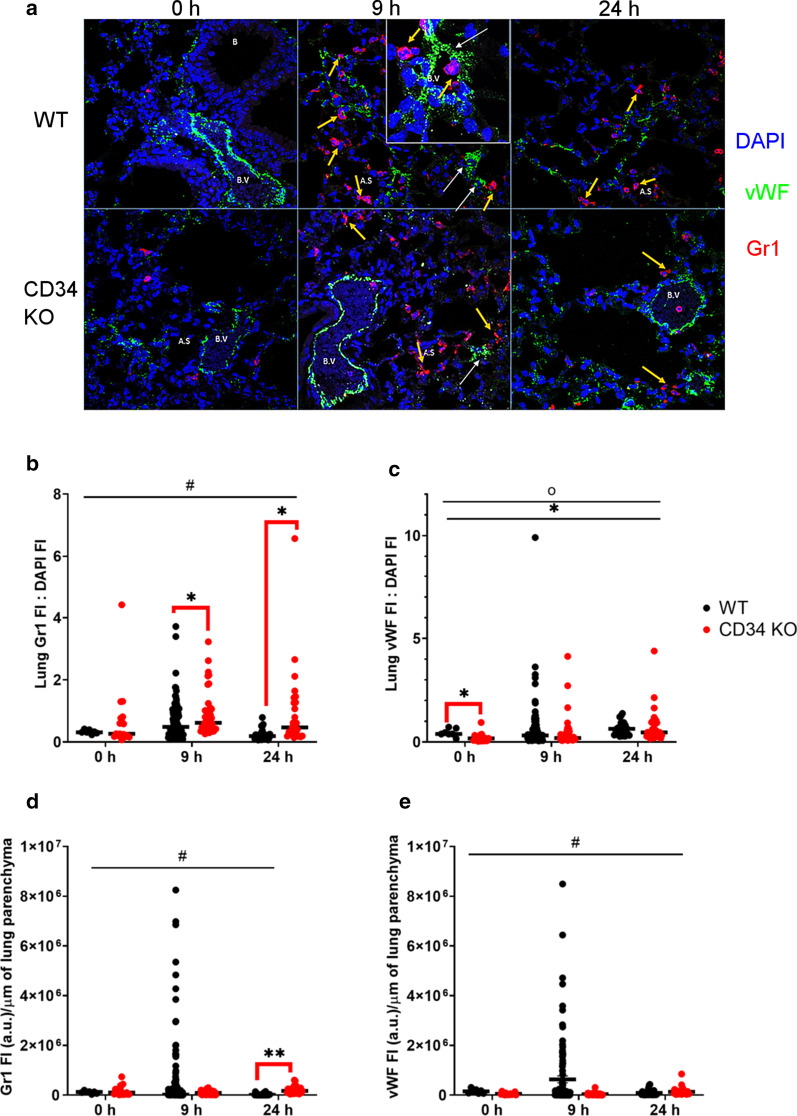
**a–e**: **a** Immunofluorescence staining in mouse lung sections: Immunofluorescence staining on mouse lungs was performed for various time points: 0 (no LPS), 9 and 24 h post-LPS in WT (WT, N = 2) and CD34 KO (N = 2) mice. Red: Gr-1, marker for polymorphonuclear cells or neutrophil granulocytes; Green: vWF, marker for endothelium and platelets; Blue: DAPI, staining for cell nucleus. Representative images from three independent experiments. Platelet aggregates can be seen (white arrows) as vWF positive (green stained) clusters in alveolar septal post-LPS exposure. B: Bronchiole; B.V.: Blood vessel, A.S.: Alveolar septum. Scale: 50 μm, inset 25 μm; Results from lung image quantification are expressed as individual values and median to describe the central value. Fluorescence intensities, for Gr1/vWF and DAPI, were quantified in a minimum of 3–5 regions from each image (N = 1–2) for WT and CD34 KO mice at 0, 9 and 24 h post-LPS exposure. **b** DAPI normalized Lung Gr1 fluorescence intensity (Lung Gr1 FI: DAPI FI); **c**. DAPI normalized Lung vWF fluorescence intensity (Lung vWF FI: DAPI FI); **d** Lung perimeter normalized Lung Gr1 fluorescence intensity (Lung Gr1 FI (a.u.)/μm of lung parenchyma); **e** Lung perimeter normalized Lung vWF fluorescence intensity (Lung vWF FI (a.u.)/μm of lung parenchyma). ^#^Shows significant interaction effect, *Shows main effect of time and o shows main mouse strain effect. The significant group differences are shown by bridged bars carrying *sign. *P* < 0.05

Immunofluorescence staining was performed with Gr-1 (red) which is a marker for poly-morphonuclear leukocytes and certain inflammatory monocytes/macrophages (Fig. 3a). We observed intravascular (within green stained vessels) adherent Gr1 positive neutrophils (red colored polymorphonuclear cells), especially at 9 h in post-LPS exposed WT mice compared to CD34 KO mice. The WT mice lungs showed brighter (p < 0.01, Additional file 1: Fig. S2a) and discrete (*p* < 0.01, Additional file 1: Fig. S2c) Gr1 positive cells at 9 h when compared to corresponding CD34 KO lungs. By 24 h, WT mice showed lower Gr1 fluorescence intensity per DAPI stained nucleated cell (p < 0.01) or lung perimeter (*p* < 0.01) than 9 h post-LPS exposure (Fig. 3b, d). CD34 KO mice show a different pattern of Gr1 positive cells, especially prevalent in alveolar septa at 9 and 24 h post-LPS exposure (Fig. 3a, b, d). Under homeostasis, WT lungs showed higher but diffuse and dim vWF fluorescence intensity, which was in contrast to discrete and bright but lower total vWF expression in CD34 KO lungs, when the fluorescence intensity was normalized for DAPI or nucleated cells (p < 0.05, Fig. 3c, Supplementary Fig. 2b, d). The alveolar staining for vWF (platelet aggregates) was not prominent in the CD34 KO mice at 9 h post-LPS exposure time-points, when normalized for lung perimeter (*p* < 0.01 and compared to 24 h, Fig. 3e). The IF quantification revealed increased Gr1:DAP1 in CD34 KO compared to WT mice at 9 h (*p* < 0.05) and 24 h (*p* < 0.01) post-LPS treatment (Fig. 3b).

### CD34 KO mice show reduction in LPS-induced alveolar protein exudation when compared to WT mice

**Fig. 4:**
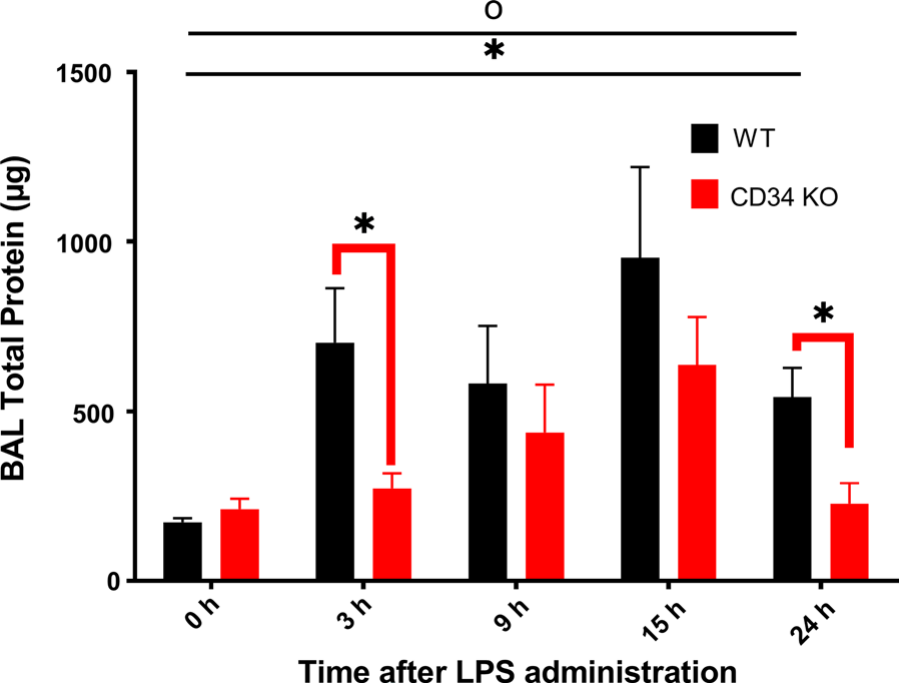
BAL Total Protein (μg) estimation assay at various time points: 0-h (no LPS; WT, N = 7; CD34 KO, N = 7), 3 h (WT, N = 6; CD34 KO, N = 6), 9 h (WT, N = 6; CD34 KO, N = 5), 15 h (WT, N = 6; CD34 KO, N = 6) and 24 h (WT, N = 6; CD34 KO, N = 6) post-LPS treatment. Main effects of time-point (*) and genotype (°) were significant. Significant group differences are shown by bridged bars carrying *Experiment was performed in duplicates. All results are expressed as mean ± SE. *P* < 0.05

Two-way ANOVA analysis indicated that main effects of time-point post-LPS challenge (*p* < 0.01) and mouse strain (*p* = 0.01) on BAL protein were significant (Fig. 4). This implied that the intranasal instillation of LPS induced time-dependent changes in pulmonary vascular permeability. As shown in Fig. 4, WT mice have a higher total protein content in the BAL supernatant at 3 h (*p* = 0.05) and 24 h (*p* = 0.01) post-LPS administration compared to the CD34 KO mice (*p* = 0.05) pointing towards an attenuated vascular permeability or epithelial protein secretion in the CD34 KO compared to WT mice in response to LPS.

### CD34 KO mice show delayed development of lung pathology upon LPS challenge compared with the WT mice

**Fig. 5:**
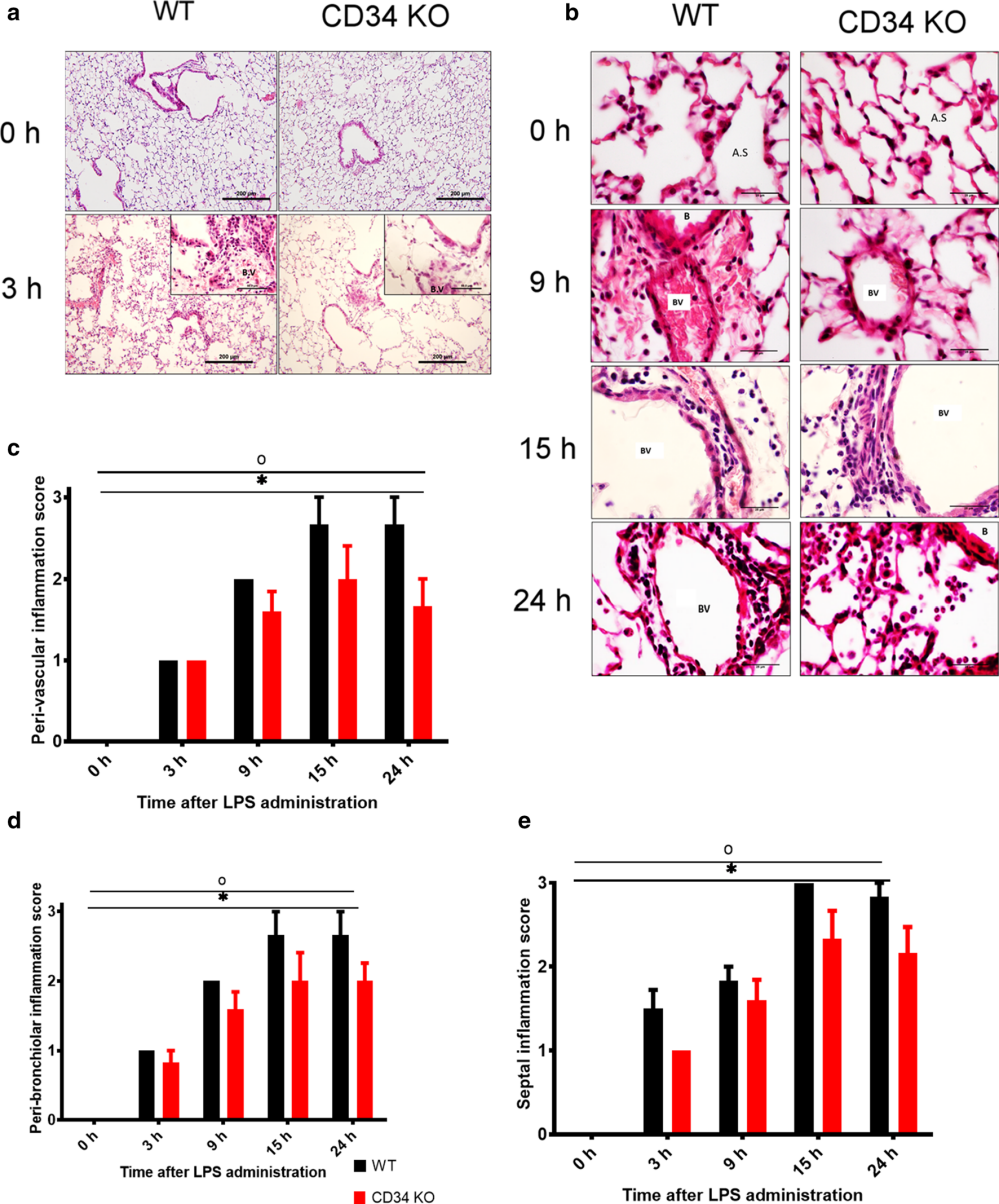
**a**–**e**:** a **Lung H&E Histology (0, 3 h) and** b** (0, 9, 15, 24 h: Representative images from H&E stained perfused left lung from 0-h (no LPS; WT, N = 7; CD34 KO, N = 7), and 3 h (WT, N = 6; CD34 KO, N = 6) post-LPS treatment. Note at 24 h, there are leukocytes adhering to the large blood vessel (BV) despite perfusion of lungs; alveolar infiltration near a bronchus (B) is significant in both strains showing dissemination of inflammation. Scale bar: a: 200 μm, inset 50 μm; b: 25 μm; **c**–**e** Histological Scoring of Inflammation in lung sections:** c** Perivascular, **d** peribronchiolar and **e** septal inflammation scores from H&E stained left lung. For perivascular, peribronchial and septal inflammation scores, interaction effect was non-significant but main effect of time-point (*) and genotype (°) were significant. No difference in perivascular, peribronchiolar, and septal inflammation score in WT vs CD34 KO at any of the time-points

H & E staining was performed on the lung samples from time points 0, 3, 9, 15 and 24 h. Lungs were congested, and perivascular space was infiltrated with leukocytes as shown at 3 h in WT mice compared to CD34 KO mice (Fig. 5a). At 9, 15 and 24 h post-LPS administration, lungs in the WT mice were more congested and had peri-vascular infiltration of leukocytes compared with the CD34 knock-out mice (Fig. 5b). The lungs obtained at 24 h post-LPS challenge showed peribronchial inflammation and many leukocytes adhering to the endothelium of large blood vessels even though these lungs were subjected to vascular perfusion. Semi-quantitative scoring of lung inflammation was also performed on H & E stained lung sections (Fig. 5c–e). Two-way ANOVA with Dunnett’s multiple comparisons test showed that scores for peri-vascular inflammation (Fig. 5c), peri-bronchial inflammation (Fig. 5d) and septal inflammation (Fig. 5e), the main effects of time-point (p < 0.0001) and mouse strain (p < 0.01) were significant. This indicates that LPS induced time-dependent changes in peri-vascular, peri-bronchiolar, and septal inflammation in both groups. However, no differences were observed between WT and CD34 KO groups for peri-vascular, peri-bronchiolar and septal inflammation scores (FigS. 5c–e).

### CD34 KO mice show reduced concentrations of cytokines and chemokines in BAL upon LPS challenge compared with the WT mice

**Fig. 6:**
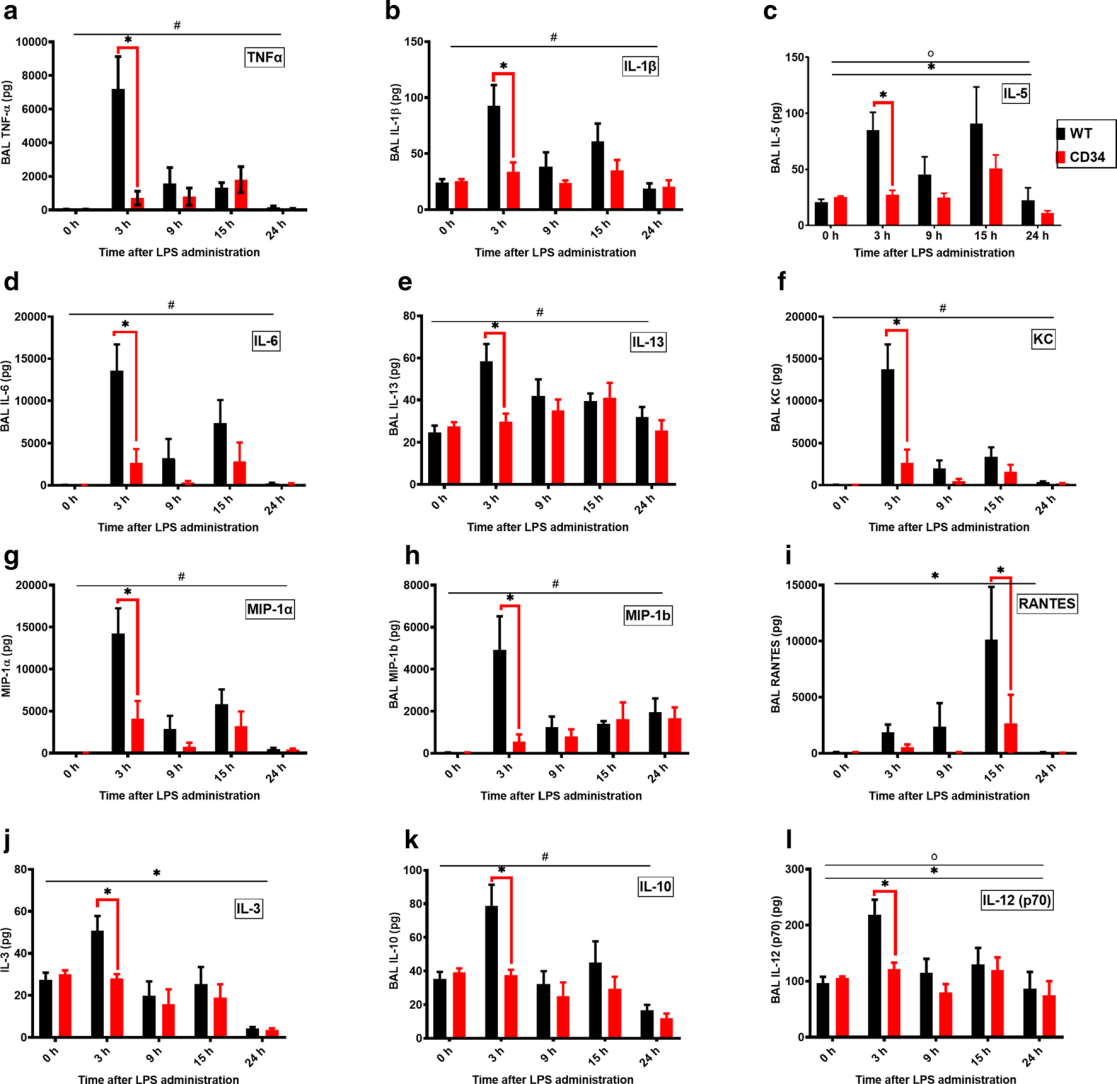
**a**–**l**Cytokine content in BAL fluid at various time points: 0-h (no LPS; WT, N = 7; CD34 KO, N = 7), 3 h (WT, N = 6; CD34 KO, N = 6), 9 h (WT, N = 6; CD34 KO, N = 5), 15 h (WT, N = 7; CD34 KO, N = 6) and 24 h (WT, N = 6; CD34 KO, N = 6) post-LPS treatment. # indicates significant interaction effect, *Indicates significant time point effect and °Indicates significant mouse-strain effect. Group differences are shown by bridged bars with *P < 0.05

The 2-way ANOVA revealed significant effect of time with LPS treatment in the cytokines (Additional file 1: Table S1). We however focused directly on the comparison between the WT and CDKO mice. There were no differences in any of the concentrations of inflammatory proteins between normal WT and CD34 KO mice. WT mice had significantly higher levels of TNF-α (*p* < 0.0001; Fig. 6a), IL-1β (*p* = 0.0008; Fig. 6b), IL-5 (*p* < 0.05, Fig. 6c), IL-6 (*p* = 0.0004; Fig. 6d), IL-13 (*p* < 0.05; Fig. 6e) at 3 h of LPS treatment compared to CD34 KO.

LPS induced significantly higher levels of chemokines KC (*p* < 0.0001; Fig. 6f), MIP1α (*p* < 0.0001; Fig. 6 g) and MIP1β (*p* < 0.0001; Fig. 6 h) in WT mice compared to CD34 KO mice at 3 h and of RANTES (*p* = 0.0403; Fig. 6i) at 15 h of the treatment.

The data showed significantly higher levels of IL-3 (Fig. 6j), IL-10 (*p* = 0.0017; Fig. 6 k) and IL-12 (p70) (Fig. 6 l) in WT mice compared to CD34 KO mice at 3 h of LPS treatment. Out of all the cytokines, GM-CSF was the only one that had significantly higher levels in LPS-treated CD34 KO mice compared to WT mice at 15 h (Additional file 1: Table S1).

There were no differences between BAL levels of LPS-treated WT and CD 34KO mice for any of the time points for IL-12 (p40), IL-1α, IL-2, IL-4, IL-9, IL-17 and MCP-1 (data not shown, Additional file 1: Table S1).

## Discussion

In this study, we have used a mouse model of lung inflammation induced with intranasal *E. coli* O55:B5 LPS (50 µL/mice) to study the role of CD34 in acute lung inflammation. This model does not replicate the inflammation induced by the instillation of live whole pathogen but also lacks the confounding variable of microbial replication in the study of mechanisms of lung inflammation. CD34 KO mice have been utilized previously in studying various inflammatory conditions as well as in one study of ALI in a murine bleomycin model [29]. Our data show reduced early (3 h) lung inflammation as indicated by reduced alveolar permeability, neutrophil migration and BAL cytokines in CD34 KO mice, which is in contrast to the effects seen at later time points of 36 and 72 h post-LPS treatment [30]. Inflammation, as we know, is accompanied by waves of inflammatory mediators released from various cells in the bone marrow or extra-medullary pools. Thus, the apparent differences in the phenotypes of lung inflammation are likely due to the release of cells and cytokines from these sites at various time points during the course of inflammation. It is possible that increase in neutrophil migration at later stages may be part of the compensatory response to their earlier reduced recruitment due to CD34 deficiency. Because our observations from samples collected at static time points and not through continuous real-time observations, there is a need for further caution in interpretation of the data from this study.

The recruitment of neutrophils and increase in alveolar permeability are hallmarks of acute lung inflammation. While there was reduced alveolar permeability at 3 h and 24 of LPS treatment in CD34 KO mice compared to WT mice, there was no difference in the numbers of neutrophils in the BAL fluid. There are earlier data on the dissociation between alveolar permeability and neutrophil migration into the lung [31]. Previous studies show an increase in vascular permeability following administration of CD34 + stem cells [32]. The higher levels of GC-CSF in LPS-challenged CD34 KO mice observed in our study may also be protective against alveolar permeability [33]. Although further studies are needed using methods such Evans Blue extravasation, the current data point out that loss of CD34 strengthens the alveolar barrier resulting in reduced permeability thus indicating that CD34 promotes alveolar permeability in inflamed lungs.

The hallmark of lung inflammation is the recruitment of neutrophils into the alveolar spaces and the lung tissues. Our results did not show significant difference between the total leukocyte counts in BAL at any of the post-LPS administration time-points in WT mice compared with CD34 KO mice. This observation is similar to the one obtained in the bleomycin model study in CD34 KO mice [29]. Similarly, no difference was observed in the alveolar macrophage counts between WT and CD34 KO group at any of the post-LPS administration time-points. The number of leukocytes in the BAL samples account for a small proportion of the blood cells and the migration of these cells may not significantly impact their numbers in the blood. Also, it is well known that there is a marginated pool of neutrophils in lung capillaries which don't contribute to the cell numbers in peripheral blood as was observed in our experiments. Those cells that had migrated into the alveoli of the mice upon LPS-administration and subsequently washed off with BAL procedure. BAL however does not remove all of the alveolar cells such as macrophages which may be adhered to alveolar epithelium. Furthermore, most neutrophils get trapped in the vasculature or interstitial space of inflamed lungs [34]. Lung MPO assay in our experimental design, is thus an indicator of the number of adherent neutrophils left in the lung after lavage and vascular lung perfusion. Interestingly, the results show that the lung MPO content was significantly higher at 3 h of LPS treatment in WT mice compared with CD34 KO mice. CD34 KO mice showed a delayed rise in MPO, starting at 9 h and continues through 15 h after LPS exposure. Furthermore, immunofluorescence staining results show prominent alveolar staining with Gr-1, a marker for polymorphonuclear leukocytes and a subset of inflammatory monocytes or macrophages, in CD34 KO mice at later time-points (9 and 24 h). This later increase in neutrophils in the lungs of CD34 KO mice is in agreement with the data obtained at 36 h post-LPS treatment [30]. We also noted a lower vWF alveolar septal expression in CD34 KO mice at baseline and post-LPS exposure, which points to a plausible suppression of LPS induced platelet activation in CD34 KO mice. Platelet activation is essential for heterotypic leukocyte adherence to pulmonary capillaries in the early stages of lung inflammation and a potential defect in platelet response in CD34 KO mice has been reported earlier [30] 35. The lower vWF expression may also explain slower decline in peripheral blood platelets at 3 h of LPS treatment in CD34 KO mice compared to WT mice. Taken together, our data show a slower early response in neutrophil recruitment likely an outcome slower platelet response in LPS-challenged CD34 KO mice.

The secretion of cytokines that promote or suppress inflammation occurs during clinical endotoxemia and also observed following experimental LPS challenge. The constitutive lung cells such as airway epithelial cells and macrophages along with those recruited into the lung engage, identify and respond to LPS via TLR4 leading to mobilization of transcription factors such as NF-kB and production of cytokines [36]. Therefore, the cytokines observed in BAL are contributed by a very diverse population of cells. We found lower concentrations of IL-1β, TNF-α, IL-6, IL-5, IL-10 and IL-13 at 3 h of LPS treatment in BAL of CD34 KO mice compared to WT mice. Our results are similar to previous studies, where TNF-α levels begin to rise as early as 30 min post intratracheal LPS challenge and peak at 1 h in WT mice, the levels remain high for up to 4 h [37, 38]. IL-6 activates leukocytes, and IL-6 levels correlate with severity of ARDS [39]. IL-6 levels in rats have been shown to peak within 6 h post-intratracheal LPS challenge and it was not detected in BAL until an hour post-LPS challenge [38]. IL-10 has been associated with high mortality rate in ARDS [39] and we have earlier reported its expression along with pro-inflammatory cytokines in a model of sepsis [40]. Mice do not express IL-8 but they have KC chemokine, which is a mouse homologue of human IL-8 and is induced by pro-inflammatory cytokines TNF-α, IL-6, IFN-γ and IL-1β, and acts as a neutrophil chemoattractant [41, 42]. The significantly higher expression of KC, MIP1α and MIPβ in WT mice at 3 h of LPS treatment may be critical in higher migration of neutrophils as indicated MPO levels in these mice. MIP-1α and β are pro-inflammatory chemokines released by monocytes and macrophages and induce neutrophil chemotaxis in mice [43]. RANTES is actively involved in recruitment of leukocytes such as macrophages, basophils, and eosinophils to the site of inflammation [44]. In addition stimulation of chemokine production, inflammatory cytokines such as TNF-α, IL-6, and IL-1β, enhance expression of adhesion molecules to recruit inflammatory cells. As it is now well understood that no single cytokine regulates cascade of inflammation, our data collectively also point to complex actions, as expectd, of many cytokines in differentiating the CD34-mediated differences in lung inflammation between WT and CD34KO mice.

## Conclusion

The data show reduction in alveolar permeability, neutrophil migration into the lung and many inflammatory cytokines in LPS-treated CD34 KO mice. This points out a role for CD34 in acute lung inflammation. The study also raises some interesting questions on the role of platelets in CD34 KO mice in influencing recruitment of neutrophils.

## Supplementary Information

**Additional file 1.** **Figure S1.** Experiment Design. **Figure S2.** a-d: Lung immune-fluorescence quantification. **Table S1**. BAL cytokine p-level statistics.

## Data Availability

The data and any material can be shared.

## Abbreviations

BAL: Broncho-alveolar Lavage
IL: Interleukin
KO: Knock-out
LPS: Lipopolysaccharide
MCP: Monocyte Chemoattractant Protein
MIP: Macrophage Inflammatory Protein
MPO: Myeloperoxidase
RANTES: Regulated upon Activation, Normal T Cell Expressed and Presumably Secreted
vWF: Von Willebrand Factor
TNFα: Tumor Necrosis Factor -alpha
