# Supplementary material for: Lack of CD34 delays bacterial endotoxin-induced lung inflammation
Source: Respir Res. 2021 Feb 25;22:69. doi: 10.1186/s12931-021-01667-2 (PMC7908703; doi:10.1186/s12931-021-01667-2)
Supplement: Supplementary file 1 — Additional file 1. Figure S1. Experiment Design. Figure S2. a-d: Lung immune-fluorescence quantification. Table S1. BAL cytokine p-level statistics. [file 12931_2021_1667_MOESM1_ESM.pdf]

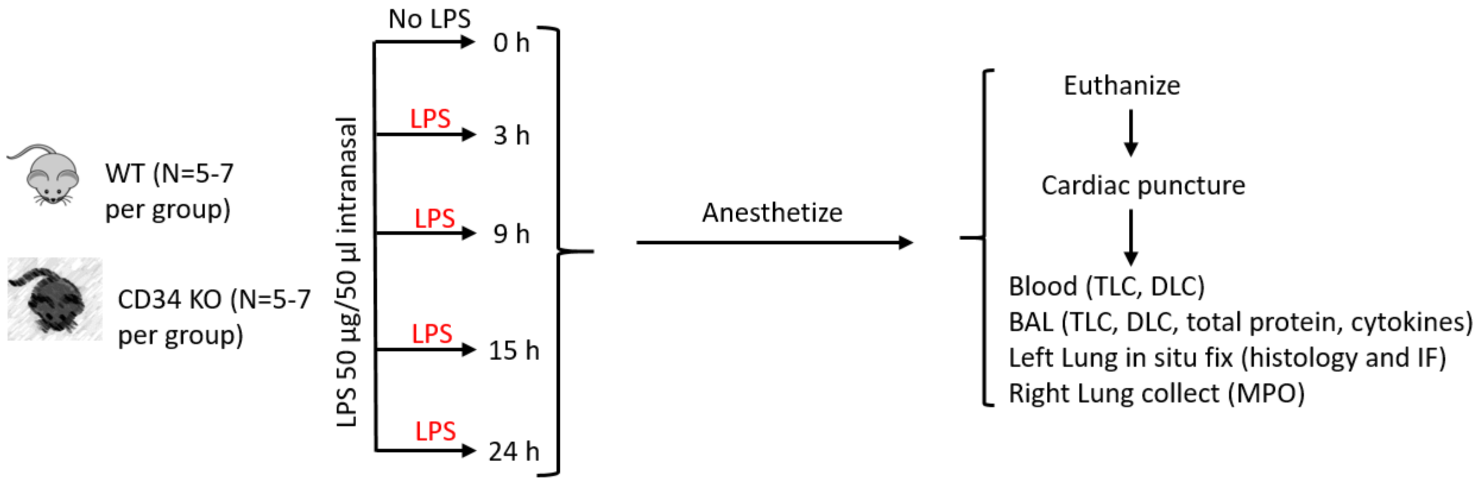
No LPS
0 h
LPS
3 h
WT (N=5-7
per group)
LPS
9 h
CD34 KO (N=5-7
per group)
LPS
15 h
LPS
24 h
LPS 50 µg/50 µl intranasal
Anesthetize
Euthanize
Cardiac puncture
Blood (TLC, DLC)
BAL (TLC, DLC, total protein, cytokines)
Left Lung in situ fix (histology and IF)
Right Lung collect (MPO)

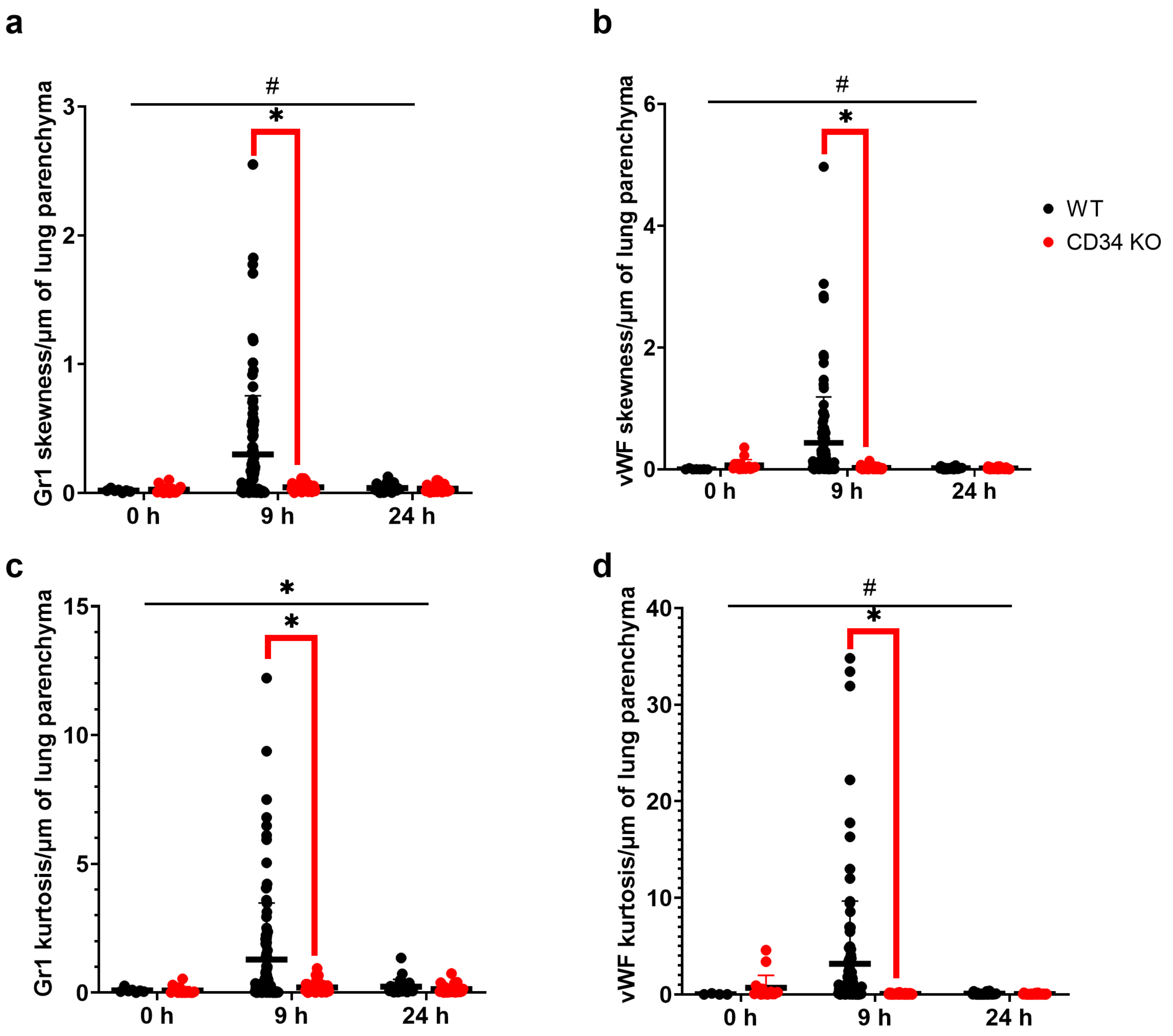
a
Gr1 skewness/µm of lung parenchyma
0 h
9 h
24 h
b
vWF skewness/µm of lung parenchyma
WT
CD34 KO
c
Gr1 kurtosis/µm of lung parenchyma
d
vWF kurtosis/µm of lung parenchyma

S. No.	Cytokine	Interaction	Time effect	Genotype effect
1	**Mo IL-1a**	>0.05	0.0001	>0.05
2	**Mo IL-1b**	0.0235	0.0003	0.0039
3	**Mo IL-2**	>0.05	<0.0001	>0.05
4	**Mo IL-3**	>0.05	<0.0001	0.0631
5	**Mo IL-4**	>0.05	<0.0001	>0.05
6	**Mo IL-5**	>0.05	<0.0001	0.0358
7	**Mo IL-6**	0.0197	<0.0001	0.0019
8	**Mo IL-9**	>0.05	<0.0001	>0.05
9	**Mo IL-10**	0.0423	<0.0001	0.0083
10	**Mo IL-12(p40)**	>0.05	0.0018	0.001
11	**Mo IL-12(p70)**	>0.05	0.0017	0.039
12	**Mo IL-13**	0.0303	0.0036	0.0288
13	**Mo IL-17**	>0.05	<0.0001	>0.05
14	**Mo Eotaxin**	>0.05	0.0573	>0.05
15	**Mo G-CSF**	>0.05	0.0008	0.0311
16	**Mo GM-CSF**	0.0142	0.0059	>0.05
17	**Mo IFN-g**	>0.05	>0.05	>0.05
18	**Mo KC**	<0.0001	<0.0001	0.0003
19	**Mo MCP-1**	>0.05	0.0019	>0.05
20	**Mo MIP-1a**	0.066	<0.0001	0.0021
21	**Mo MIP-1b**	0.0034	0.0013	0.018
22	**Mo RANTES**	>0.05	0.009	>0.05
23	**Mo TNF-a**	<0.0001	<0.0001	0.0017
